# Everyday Digital Literacy Questionnaire for Older Adults: Instrument Development and Validation Study

**DOI:** 10.2196/51616

**Published:** 2023-12-14

**Authors:** JiYeon Choi, Seongmi Choi, Kijun Song, Jiwon Baek, Heejung Kim, Mona Choi, Yesol Kim, Sang Hui Chu, Jiyoung Shin

**Affiliations:** 1 Mo-Im Kim Nursing Research Institute College of Nursing Yonsei University Seoul Republic of Korea; 2 Institute for Innovation in Digital Healthcare Yonsei University Seoul Republic of Korea; 3 College of Nursing and Brain Korea 21 FOUR Project Yonsei University Seoul Republic of Korea

**Keywords:** aging, older adults, digital literacy, instrument, validation, psychometrics, European Commission’s Digital Competence framework

## Abstract

**Background:**

The need for digital literacy in aging populations is increasing in the digitalizing society. Digital literacy involves the identification, evaluation, and communication of information through various digital devices or relevant programs.

**Objective:**

The aims of this study were to develop an Everyday Digital Literacy Questionnaire (EDLQ), a digital literacy assessment scale, and subsequently evaluate its psychometric properties using a population of community-dwelling older adults in South Korea.

**Methods:**

The EDLQ was developed using an instrument development design. A nationwide survey was conducted, and the study included 1016 community-dwelling older adults (age ≥60 years). To evaluate the psychometric properties, the participants were randomly divided into 2 groups (n=508 each), and the internal consistency (Cronbach α and McDonald ω), structural validity (exploratory factor analysis and confirmatory factor analysis), hypothesis-testing construct validity using the eHealth Literacy Scale (eHEALS), and measurement invariance were analyzed.

**Results:**

Among the initial 30 items of the EDLQ, 22 items with a 3-factor solution had a total explained variance of 77%. The domains included “information and communication” (9 items), “content creation and management” (4 items), and “safety and security” (9 items). Confirmatory factor analysis was conducted with this 3-factor solution (*χ*^2^_206_=345.1; normed *χ*^2^_206_=1.7; comparative fit index=0.997; Tucker-Lewis index=0.997; root-mean-square error of approximation=0.036; standardized root-mean-square residual=0.050; composite reliability=0.903-0.959; average variance extracted=0.699-0.724; *R*^2^=0.616-0.773). Hypothesis-testing construct validity with the eHEALS revealed a strong correlation (*r*=0.75). Cronbach α and McDonald ω coefficients were .98 and 0.98, respectively. The fit indices for measurement invariance, including the configural, metric, and scalar invariance models, demonstrated a satisfactory fit to the data. Our findings suggest that the psychometric properties of the 22-item EDLQ are valid and reliable for assessing digital literacy among older Korean adults.

**Conclusions:**

In this study, we developed a digital literacy measure with strong psychometric properties that made it suitable for assessing the digital literacy of community-dwelling older adults in Korea. To broaden its applicability, however, further assessment of its feasibility for use with different languages and cultures is necessary. Moreover, more empirical research on digital literacy and related factors in older adults can facilitate the development of personalized digital health care services and educational interventions in the digital society.

## Introduction

Advancements in digital technology have great potential for promoting healthy aging among older adults. Previous studies have reported the role of digital technology in preserving [1] and assisting with functional independence [2], preventing injury [3], fostering social connectedness [4], and contributing to the treatment of mental health conditions [5]. Overcoming barriers associated with age-related health changes, including chronic illness, reduced mobility, and a shift in social dynamics, using digital technology can empower older adults to maintain their health and quality of life [6].

The number of older adults using technology has increased gradually; however, a substantial difference persists between age groups in digital technology use. According to a recent report by the Organization for Economic Cooperation and Development, in 2019, internet use was reported in only 58% of adults aged 55-74 years, whereas it was reported in 95% of individuals aged 16-24 years [7]. Aging-related functional decline and psychosocial factors have been identified as major barriers to internet use and technology adoption among older adults [1,8-11].

Understanding digital literacy among older adults is crucial for fostering their successful engagement with digital technology. Therefore, the attitudes, beliefs, and cognitive factors unique to older adults need to be characterized [12,13]. Digital literacy refers to the ability to read and understand information in digital formats, including hypertext or multimedia [14]. It covers more than just specific skills and encompasses ideas, mindsets, and the ability to comprehend and navigate digital information formats [15]. However, the definition of digital literacy is evolving and has been used interchangeably with other related terms, including information literacy, media literacy, computer literacy, information and communication technology literacy, network literacy, and e-literacy [15]. Therefore, the Digital Competence (DigComp) framework was introduced to standardize the key elements of digital literacy [16]. The DigComp framework comprises 5 domains for assessing knowledge, skills, and attitudes related to digital competence across different age groups, sex, and education levels [17].

As the definition of digital literacy is ever-evolving, reassessing and enhancing existing digital literacy measures are important. Existing measures have primarily focused on searching information and communication aspects while neglecting other critical components such as content creation or safety [13]. Furthermore, the evaluation of digital capabilities based on the DigComp framework has predominantly targeted students, teachers, and adults aged 65 years or younger [17-19]. Thus, the framework has not been validated in older adults [19]. Accordingly, the aim of this study was to develop and validate an Everyday Digital Literacy Questionnaire (EDLQ) using the DigComp framework.

## Methods

In this study, the EDLQ was developed using the 4 phases of instrument development and validation (conceptualization, item development, content validation, and a field survey) to evaluate its psychometric properties.

### Ethical Considerations

In accordance with the Helsinki Declaration, all procedures in this study have been reviewed and approved by the Institutional Review Board of Yonsei University (approval 4-2022-0396).

### Phase I: Conceptualization

This step involved identifying the concept and scope of digital literacy using the DigComp framework comprising five domains: (1) information literacy, (2) communication and collaboration with others, (3) content creation and editing, (4) safety and security, and (5) problem-solving [16]. Based on the DigComp, in this study, digital literacy was defined as the competence to find, evaluate, create, share, and interact independently and safely using digital devices. The scope of digital devices includes desktops and laptop computers, mobile phones, tablets, e-books, and wearable devices. The digital activities assessed were messaging, information search, and digital social activities including emailing, blogging, e-learning, and application use.

### Phase II: Item Development

The development of the preliminary items involves multiple steps. First, we referred to the 25-item self-assessment grid from the European Commission that outlined the foundation level of digital literacy. This step was done to ensure the inclusion of relevant items aligned with the digital competence levels of older adults [1,8,16]. Additionally, we conducted a systematic review of previous studies that assessed digital literacy among older adults. We identified instruments such as the eHealth Literacy Scale (eHEALS), Unified Theory of Acceptance and Usage of Technology, Loyd-Gressard Computer Attitude Scale, and Technology Acceptance Model. These instruments provided valuable insights, and we referred to them during the item development. Furthermore, we conducted qualitative interviews with older adults residing in the urban community before developing the items. These interviews aimed at collecting additional information on digital technology use and literacy. Following the distribution of flyers at the local senior community center and the use of snowball sampling, 14 participants (aged ≥65 years) consented and completed semistructured interviews for 25-51 minutes. All participants were remunerated after completing the interview. The qualitative content analysis was performed to specify the experiences of Korean older adults with the use of digital devices and to examine contextual factors using the 5 domains of the DigComp framework (Baek et al, unpublished data, September 2023). By integrating insights from the self-assessment grid, validated instruments, and qualitative interviews, we developed a set of 35 preliminary items.

### Phase III: Content Validation

We invited 7 external experts representing diverse disciplines, including health informatics, public health, medicine, sociology, social work, and measurements. The panel of multidisciplinary experts agreed to participate in content validation, and all of them were remunerated for their time. Each expert was asked to evaluate the preliminary item pool based on 3 criteria: relevance (the extent to which the items aligned with the definition of digital literacy across the 5 domains), clarity (the clarity and conciseness of the items), and comprehensiveness (whether the items adequately covered all 5 domains of digital literacy). Relevance and clarity were assessed using a 4-point Likert scale (1=very inappropriate, 2=inappropriate, 3=appropriate, and 4=very appropriate), while comprehensiveness was evaluated using open-ended questions to encourage suggestions.

To examine the content validity, we used the item-level content validity index (I-CVI) and the scale’s content validity index (S-CVI) [20]. The I-CVI measured the proportion of panel members who rated an item as “appropriate” or “very appropriate,” while the S-CVI represented the average of the I-CVI values [20]. Our predefined criteria for excellent content validity required an I-CVI of ≥0.78 and an S-CVI/AVE (average variance extracted) of ≥0.90, which indicated consensus among at least 3 expert panel members [20].

### Phase IV: Field Survey

#### Study Design

A cross-sectional survey was conducted to assess the validity of the EDLQ. This survey aimed to evaluate structural and hypothesis-testing construct validity, internal consistency, measurement invariance, and floor and ceiling effects.

#### Sample and Data Collection

A total of 1016 participants were recruited nationwide from October to November 2022 using proportional stratified sampling based on region, sex, and age groups. The sampling procedure was designed to align with the registered population of South Korea in June 2022. The inclusion criteria for the sample were (1) individuals aged ≥60 years, (2) achieving a minimum score of 22 on the Korean version of the Mini-Mental State Examination (second edition), and (3) fluency in the Korean language. For data collection, a team of 58 trained interviewers visited potential participants’ households. The interviewers provided detailed information on the study and sought consent from eligible individuals. Once participants voluntarily agreed to participate in the survey, they completed structured questionnaires on a tablet. Before the survey, all participants provided informed consent. After completing the survey, they received gift vouchers as compensation.

#### Measures

The eHEALS, a validated measure of eHealth literacy, was used as a comparator instrument to assess the construct validity of the EDLQ using a hypothesis-testing approach. Based on the prior studies [21-23], we hypothesized that participants with higher levels of digital literacy would also have higher eHEALS scores. The eHEALS comprises 10 items scored on a 5-point Likert scale. The scale score is calculated by summing all items except for the first 2, with higher scores indicating better eHealth literacy [24]. The original validation study of the eHEALS demonstrated good internal consistency, with a Cronbach α of .88 and a test-retest reliability intraclass correlation coefficient of 0.49 [24]. Construct validity analysis yielded a 1-factor solution that accounted for 56% of the total variance [24]. The Korean eHEALS was developed and validated using a sample of 180 older Korean adults. The Korean eHEALS demonstrated good internal consistency (Cronbach α=.90; item-total correlation coefficients=0.57-0.75) and a 1-factor structure that explained 77% of the total variance, supported by model fit indices [25]. In this study, Cronbach α for the instrument was .97.

#### Data Analysis

The data were analyzed using SAS for Windows (version 9.4; SAS Institute Inc) and the *lavaan* package in R (R Foundation for Statistical Computing). For the cross-validation of structural validity, the total sample was randomly divided into 2 subsamples (n=508 each) using simple random sampling in SAS. One of the subsamples (subsample 1) was used for exploratory factor analysis (EFA), while the other (subsample 2) was used for confirmatory factor analysis (CFA). The sample sizes of both subsamples met the recommended guidelines of at least 10 times the number of items for EFA and a minimum of 200 cases for CFA [26]. Chi-square test was used to compare the differences between the EFA and CFA sample groups. Prior to conducting the EFA in subsample 1, an interitem correlation matrix was generated for all items, and weakly correlated (*r*<0.30) or redundant (*r*>0.80) items were removed based on the contents of the questions [27].

To determine the suitability of subsample 1 data for EFA, the Kaiser-Meyer-Olkin (KMO) test of sampling adequacy and Bartlett test of sphericity were conducted. For factor analysis, a KMO index of at least 0.50 (ranging from 0 to 1) and a significant Bartlett test of sphericity (*P*<.05) were recommended [28]. Principal axis factoring extraction with the oblimin rotation method was used for the EFA to reduce the number of items and determine the underlying structure. Factors with eigenvalues >1 were retained, aiming to retain factors that accounted for at least 50%-60% of the variance [27]. A factor loading of ≥0.3 was considered appropriate for explaining the factor [29], but if an item had a factor loading of >0.3 for 2 or more factors, or the difference between factor loadings was not >0.1, it was considered cross-loaded [30].

CFA was performed on subsample 2 using a diagonally weighted least squares estimation. The goodness-of-fit of the CFA model was evaluated using established indices, including normed *χ*^2^_206_<3, comparative fit index (CFI)>0.95, Tucker-Lewis index>0.95, root-mean-square error of approximation (RMSEA)<0.08, and standardized root-mean-square residual (SRMR)<0.08 [31]. Convergent and discriminant validity were assessed using factor loadings, AVE, and composite reliability (CR). The AVE and CR values were calculated using model estimates based on the formula provided by Fornell and Larcker [32]. The criteria for convergent validity included estimated standard factor loadings >0.70, AVE value of each factor >0.50, and CR value of each factor >0.70, and all CR values were greater than the AVE values [33]. Discriminant validity was achieved when the AVE values for each construct exceeded the squared correlation between the constructs [32]. Hypothesis-testing construct validity of eHEALS was analyzed using Pearson correlation coefficient. Internal consistency was analyzed using Cronbach α and McDonald ω, with an acceptable cutoff value of 0.70 [34,35].

Measurement invariance was evaluated across sex, age, and education groups using multigroup CFA based on previous studies [36,37]. To meet the recommended minimum sample size requirement (≥100) for each group in the measurement invariance test [38], demographic characteristics were reclassified as follows: male (n=236) versus female (n=272), aged 60-69 years (n=332) versus ≥70 years (n=176), and below high school graduation (n=219) versus above high school graduation (n=289). Multigroup CFA was conducted using the following sequences [38]: (1) a configural invariance model (to test the equivalence of factor structure among groups), (2) a metric invariance model (to test the equivalence of the item loadings on the factors among groups), and (3) a scalar invariance model (to test the equivalence of item intercepts among groups). For measurement invariance, the proposed criterion for each step-by-step CFI change (ΔCFI) was <0.010, RMSEA change (ΔRMSEA) was <0.015, and SRMR change (ΔSRMR) was <0.030 (metric invariance model) or <0.015 (scalar invariance model) [39].

The floor and ceiling effects of the latent scores were assessed using descriptive statistics, with values of ≥30% of the lowest or highest scores on the instrument indicating floor or ceiling effects [40].

## Results

### Generated Items

In our qualitative research assessing digital literacy among community-dwelling older adults, participants provided diverse feedback regarding their experiences with digital technologies. We collected data on their proficiency in information and data literacy, communication or collaboration, and problem-solving related to device use. However, their competencies in creating digital content and ensuring safety and security while using digital technology were limited. By recognizing the importance of digital safety awareness and digital content creation abilities, we decided to include all 5 domains of the DigComp framework in the development of the EDLQ. Thus, we derived a set of 35 preliminary items for the EDLQ, encompassing the following domains: “information and data literacy” (7 items), “communication and collaboration” (8 items), “digital content creation” (8 items), “safety” (8 items), and “problem-solving” (4 items).

### Content Validity

Content validation of the initial 35 items revealed that the S-CVI/AVE values for relevance and clarity were 0.94 and 0.84, respectively. All items met the relevance criterion (≥0.78) in terms of the I-CVI. Moreover, 26 of the 35 items fulfilled the criterion for item clarity (≥0.90). Items that did not meet the clarity criterion were carefully reviewed and either eliminated or modified to improve clarity based on suggestions provided by the expert panel. As a result, a final set of 30 items was selected for inclusion in the EDLQ, representing 5 domains described in “generated items.” To ensure the linguistic accuracy and appropriateness of the selected items, they were further evaluated and fine-tuned by a linguistic expert.

### Field Survey

#### General Characteristics

Among the 1016 participants, the majority were female (n=530, 52.2%), were in the 60-69–year age group (n=666, 65.5%), were residing in nonmetropolitan areas (n=547, 53.8%), had completed education above high school graduation (n=551, 54.2%), had a spouse (n=766, 75.4%), were engaged in economic activities (n=622, 61.2%), and did not have any physical disabilities (n=967, 95.2%). No statistically significant differences were observed in the basic characteristics of subsamples 1 and 2 (Table 1).

**Table 1:** General characteristics of the study participants.

		Total (N=1016), n (%)	Subsample 1 (n=508), n (%)	Subsample 2 (n=508), n (%)	Between subsamples 1 and 2	
					Chi-square (*df*)	*P* value
**Sex**					0.8 (1)	.38
	Male	486 (47.8)	250 (24.6)	236 (23.2)		
	Female	530 (52.2)	258 (25.4)	272 (26.8)		
**Age (years)**					0.0 (1)	.90
	60-69	666 (65.6)	334 (32.9)	332 (32.7)		
	≥70	350 (34.5)	174 (17.1)	176 (17.3)		
**Residence**					0.0 (1)	.85
	Metropolitan area^a^	469 (46.2)	233 (22.9)	236 (23.2)		
	Nonmetropolitan area^b^	547 (53.8)	275 (27.1)	272 (26.8)		
**Education**					4.1 (3)	.26
	Below middle school	240 (23.6)	132 (13)	108 (10.6)		
	Below high school	225 (22.2)	114 (11.2)	111 (10.9)		
	Below college	451 (44.4)	212 (20.9)	239 (23.5)		
	College and above	100 (9.8)	50 (4.9)	50 (4.9)		
**Presence of spouse**					0.0 (1)	>.99
	Yes	766 (75.4)	383 (37.7)	383 (37.7)		
	No	250 (24.6)	125 (12.3)	125 (12.3)		
**Engaged in economic activities**					2.4 (2)	.30
	Yes	622 (61.2)	320 (31.5)	302 (29.7)		
	Used to	354 (34.8)	172 (16.9)	182 (17.9)		
	Never	40 (3.9)	16 (1.6)	24 (2.4)		
**Disability**					3.6 (1)	.06
	Yes	49 (4.8)	31 (3.1)	18 (1.8)		
	No	967 (95.2)	477 (47.0)	490 (48.2)		

^a^Metropolitan area: Seoul, Incheon, and Gyeonggi province.

^b^Nonmetropolitan area: cities and provinces other than the metropolitan area.

#### Interitem Correlation Matrix

As shown in Multimedia Appendix 1, the interitem correlation coefficients among all items in subsample 1 (EFA data set) ranged from 0.43 to 0.91 (*P*<.001). None of the item pairs had a weak correlation (*r*<0.30), while 13 item pairs had a strong correlation (coefficient>0.80). A correlation coefficient close to 1.0 indicates that the 2 items have similar meanings or provide the same information [41]. Careful examination of the content of the strongly correlated items revealed that 3 item pairs were overlapping (items 2 and 3, 21 and 22, and 28 and 29), leading to the removal of 1 item from each pair (items 3, 22, and 28).

#### Structural Validity

After conducting the EFA using the 27-item subsample 1, Bartlett sphericity test revealed a significant result, and the KMO value was 0.97, indicating excellent factorability of the data. EFA with principal axis factoring extraction and oblimin rotation resulted in a 3-factor solution (eigenvalue>1) explaining 75.6% of the variance (Multimedia Appendix 2). Of the 27 items, 26 meaningfully loaded onto 1 of the 3 factors, with factor loadings ranging from 0.38 to 0.89. However, 1 item (item 11) had cross-loading. Therefore, this item has been deleted. Subsequent EFAs (2-4) led to the removal of 1 item each (items 13, 12, and 15) based on the same criteria as the first EFA. In the fifth EFA, no cross-loading was identified, but 1 item (item 16) was deemed unsuitable for the factor with the maximum loading value and was therefore deleted. The sixth EFA, conducted with 22 items, resulted in a 3-factor solution that explained 77% of the variance (Table 2). The factors were labeled as “information and communication” (9 items), “content creation and management” (4 items), and “safety and security” (9 items).

**Table 2:** Results of internal consistency.

Instrument and domains		Cronbach α	McDonald ω
**Total EDLQ^a^ (22-item version)**		.98	0.98
	Factor 1: Information and communication	.96	0.96
	Factor 2: Contents creation and management	.91	0.91
	Factor 3: Safety and security	.96	0.96

^a^EDLQ: Everyday Digital Literacy Questionnaire.

**Figure 1:**
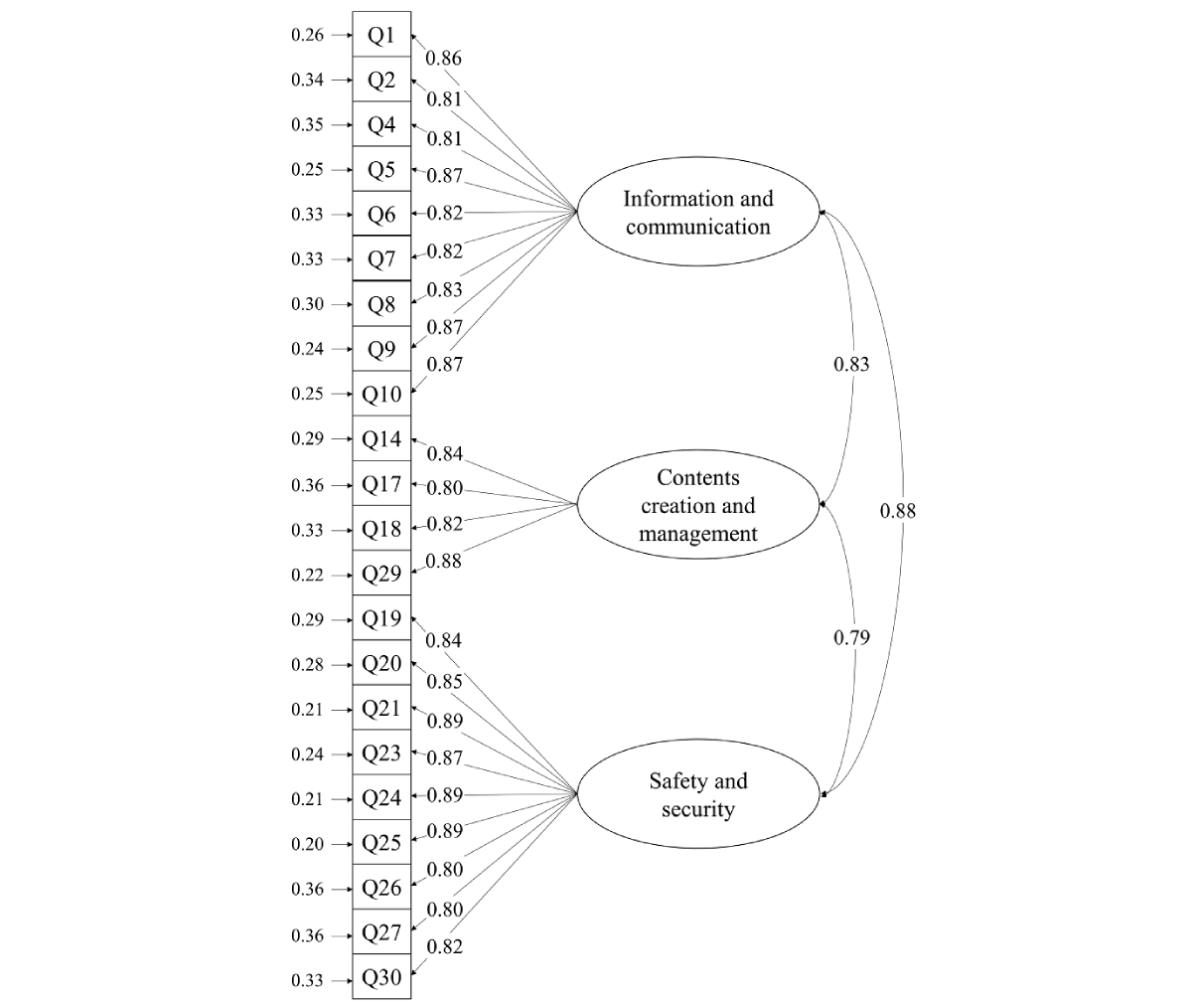
Findings of confirmatory factor analysis for the Everyday Digital Literacy Questionnaire. Q: questionnaire.

Following analysis of the EFA results, a 3-factor solution with diagonally weighted least squares estimation was used for the CFA on subsample 2. As presented in Table 3, the CFA demonstrated a favorable fit across all indices (normed *χ*^2^_206_=1.7; CFI=0.997; Tucker-Lewis index=0.997; RMSEA=0.036; and SRMR=0.050). The CR values for each factor ranged from 0.903 to 0.959, indicating satisfactory internal consistency. The AVE values for each factor ranged from 0.699 to 0.724, meeting the recommended criteria for convergent validity. The distribution of standardized factor loading values (Figure 1) further supported convergent validity with values ranging from 0.80 to 0.89. In terms of discriminant validity, the squared correlation between the pairs of factors ranged from 0.616 to 0.773 compared with the AVE values, indicating partial satisfaction with discriminant validity.

**Table 3:** Summary of fit indices in confirmatory factor analysis.

Fit indices	Recommended criterion	EDLQ^a^
Normed *χ*^2^	<3	1.7 (*df*=206)
CFI^b^	>0.95	0.997
TLI^c^	>0.95	0.997
RMSEA^d^ (90% CI)	<0.08	0.036 (0.030-0.043)
SRMR^e^	<0.08	0.050
AVE^f^ of each factor	>0.50	0.699-0.724
CR^g^ of each factor	>0.70	0.903-0.959

^a^EDLQ: Everyday Digital Literacy Questionnaire.

^b^CFI: comparative fit index.

^c^TLI: Tucker-Lewis index.

^d^RMSEA: root mean square error of approximation.

^e^SRMR: standardized root mean square residual.

^f^AVE: average variance extracted.

^g^CR: composite reliability.

#### Hypothesis-Testing Construct Validity

The correlation coefficient between EDLQ and eHEALS was 0.75 (*P*<.001). This supports the hypothesis that digital literacy positively correlates with eHealth literacy.

#### Internal Consistency

In the 22-item version of the EDLQ, the overall Cronbach α was .98, and McDonald ω was 0.98. The internal consistency of factor 1, factor 2, and factor 3 was indicated by α value of .96, .91, and .96, respectively (Table 2). These results demonstrate that the EDLQ has good internal consistency.

#### Measurement Invariance

Multimedia Appendix 3 presents the results of the measurement invariance test for the EDLQ across sex, age, and education groups. The fit indices for the configural, metric, and scalar invariance models indicated satisfactory fit to the data. Furthermore, the ΔCFI, ΔSRMR, and ΔRMSEA values for each step met the recommended criteria for measurement invariance. These findings provide evidence supporting the measurement invariance of the EDLQ across the examined groups.

#### Floor and Ceiling Effects

**Figure 2:**
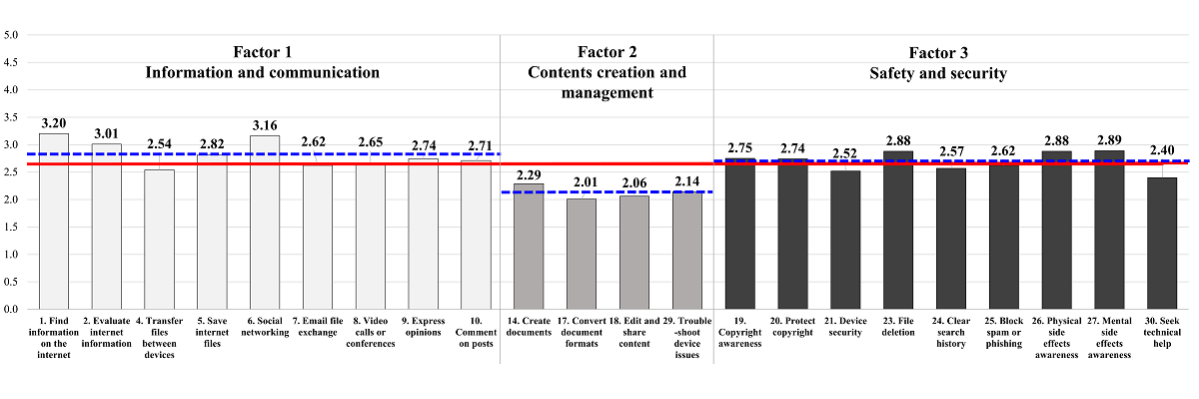
Distribution of scores for each item of the Everyday Digital Literacy Questionnaire.

In the final version of the EDLQ, participants scored the lowest on item 17 (mean 2.01, SD 1.10) and the highest on item 1 (mean 3.20, SD 1.30), as shown in Table 4 and Figure 2. In Figure 2, the red solid line presented average score for the 22 items, which was 2.65. The blue dashed line displayed the average scores for each factor, with values of 2.83, 2.13, and 2.69 for factors 1, 2, and 3, respectively. No significant floor or ceiling effects were observed in the 22-item EDLQ (floor effect 9.06%, ceiling effect 0%, and highest score of 4.91 for 0.59% of respondents), factor 1 (floor effect 11.81% and ceiling effect 2.76%), factor 2 (floor effect 27.17% and ceiling effect 0.20%), or factor 3 (floor effect 17.13% and ceiling effect 0.98%).

**Table 4:** Scores for each item in the Everyday Digital Literacy Questionnaire.

Factor	Items (abbreviated)	Values, mean (SD)
**Factor 1**		
	1. Find information I need on the Internet	3.20 (1.30)
	2. Judge whether the information from the Internet is reliable or not	3.01 (1.28)
	4. Transfer documents, photos, or video files from one device to another	2.54 (1.31)
	5. Save Internet documents, photos, or video files you find	2.82 (1.34)
	6. Exchange messages, photos, and video files through a social networking service	3.16 (1.34)
	7. Exchange documents, photos, or video files via an email	2.62 (1.42)
	8. Participate in video calls or conferences using digital devices	2.65 (1.37)
	9. Express my opinion of “like/dislike” on others’ posts	2.74 (1.33)
	10. Comment on others’ posts	2.71 (1.34)
**Factor 2**		
	14. Create a document using digital devices	2.29 (1.27)
	17. Convert document formats using digital devices	2.01 (1.10)
	18. Edit and post documents, photos, or videos created by someone else	2.06 (1.16)
	29. Independently troubleshoot issues related to device/app operation	2.14 (1.12)
**Factor 3**		
	19. Be aware of the behaviors that infringe copyright	2.75 (1.37)
	20. Protect copyright of the work from others	2.74 (1.31)
	21. Set device passwords for logging in/out	2.52 (1.35)
	23. Delete files stored on the device	2.88 (1.41)
	24. Delete my history of Internet search	2.57 (1.37)
	25. Block spam or phishing attempts on the Internet	2.62 (1.37)
	26. Be aware of the physical side effects that can result from excessive device use	2.88 (1.35)
	27. Be aware of the mental side effects that can result from excessive device use	2.89 (1.35)
	30. Know how to ask help when encountering issues during devices or app installation or operation	2.40 (1.24)

### EDLQ Scoring

The EDLQ measures each item on a 5-point Likert-type scale, ranging from 1=“strongly disagree” to 5=“strongly agree.” Calculating a total score involves summing the response scores for all 22 items on the EDLQ. The possible score in total ranges from 22 to 110, with higher scores indicating higher levels of digital literacy. The Korean version of the questionnaire is shown in Multimedia Appendix 4.

## Discussion

### Principal Results

This study developed and validated the EDLQ, an instrument for measuring digital literacy in older adults, based on the DigComp framework [16]. Overall, the study revealed satisfactory validity and reliability of the EDLQ. The results of the CFA demonstrated a good fit across the indices, and both the traditional α and the more robust ω coefficients indicated good reliability. Moreover, CFA revealed a strong correlation among the 3 factors (*r*=0.83, 0.79, and 0.88, respectively). Further exploration of the relationships among the 3 factors confirmed satisfactory convergent validity, while discriminant validity was only partially supported. These results are attributed to the nature of interfactor redundancy or cross-reference that occurs in the mechanism of real-world activities [16]. Furthermore, although the ceiling and floor effects met the recommended criteria, the floor effect of factor 2 was relatively high (27.17%). Since the EDLQ measures the foundation level of digital competence, as presented by the DigComp [16], this result may be related to the breadth of digital device use by older adults. The majority of older adults use the internet only to maintain family and social connections and access information about health and daily activities [11,42,43]. However, in older adults, developing and sharing digital content may result in meeting their psychosocial needs, such as developing their identity in older adulthood and fostering self-expression [44]. Therefore, it is important to identify and enhance older adults’ digital capabilities by measuring their proficiency in content creation, which has been overlooked by existing digital literacy instruments [12,13].

The measurement invariance of the EDLQ was satisfactory across sex, age, and education groups. Thus, the EDLQ measures the same construct regardless of sex, age, and education groups [45], and it can be used practically to measure the digital literacy of older adults with various characteristics and to compare groups.

Similar to previous studies [21-23], this study also revealed a strong correlation (*r*=0.75) between digital and eHealth literacy. Traditional literacy, information literacy, media literacy, and computer literacy, as measured by the EDLQ, are important subfactors that constitute eHealth Literacy [46]. Increasing the digital literacy of older adults will ultimately play an important role in enhancing their digital health care capabilities.

While the eHEALS is the most frequently used digital literacy measurement tool for older adults, it does not measure the ability to create digital content [13]. The Mobile Device Proficiency Questionnaire includes all 5 literacy elements, but it overlooks the protective aspects of digital literacy, such as digital identity and health [47]. The EDLQ distinguishes itself from existing tools by measuring all 5 domains of digital competence (information and data literacy, communication and collaboration, digital content creation, safety, and problem-solving) suggested by the European Commission.

**Figure 3:**
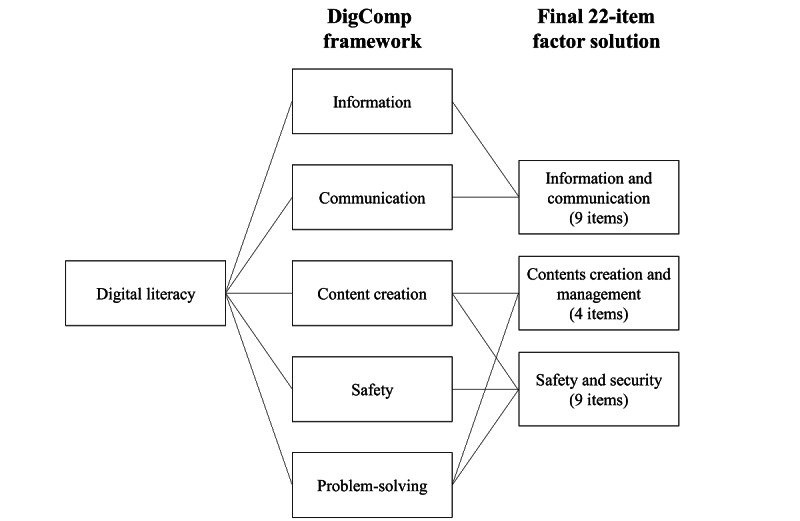
Evolution of the conceptual framework of digital literacy. DigComp: Digital Competence.

In this analysis, EFA produced a revised 3-factor solution for the EDLQ, leading to modifications in the 5 domains of the DigComp framework (Figure 3). Specifically, the domains of “information” and “communication” were combined into a single factor, while the domains of “contents creation,” “safety,” and “problem-solving” were divided into 2 distinct factors. Notably, the items that assessed copyright and licenses, originally categorized under the domain of “contents creation” in the DigComp framework, were now grouped under the domain of “safety” in this study. This change may be attributed to the intricate nature of digital activities with the intersection of different technologies. Although each domain of the DigComp framework has unique characteristics, there are points of overlap and interconnections between them [16]. The domains of “safety” and “problem-solving,” in particular, have a transversal nature that applies to various digital activities [16]. Content creation encompasses multiple elements of problem-solving, such as content development, integration, reconstruction, and programming, and is closely related to safety and copyright, making it difficult to clearly separate domains in practice.

### Limitations

This study has a few limitations. First, the EDLQ is a self-report measure; therefore, it is limited in its ability to perfectly capture objective proficiency. Second, although the EDLQ was developed for older adults, interpretation of the results is limited because the DigComp framework referenced in its development is not specific to older adults. To better understand the digital literacy of older adults, it is recommended to apply the EDLQ to different age groups in a follow-up study and compare the results. Third, the criterion validity of the EDLQ has not been tested because digital literacy is a dynamic concept that is rapidly evolving, and there is no gold standard for assessment [48]. Fourth, this study was designed cross-sectionally; therefore, the test-retest reliability of the EDLQ was not confirmed. In subsequent studies, in-depth reliability should be ensured by measuring the temporal stability of the same individual. Finally, the EDLQ has only been psychometrically tested in older Korean adults; therefore, cross-national or cross-language validations are needed to confirm its cultural invariance.

### Conclusions

As digital technology continues to advance rapidly, the concept of digital literacy is constantly evolving with it. We developed a digital literacy measure, the EDLQ, that incorporates the latest definitions of digital literacy and validated its psychometric properties. The EDLQ holds potential for application in both research and practice settings, particularly for evaluating the digital literacy of community-dwelling older adults living in South Korea. However, to fully broaden the scope of its future use, further examinations to assess its applicability to different languages and cultures are warranted. Further empirical research on the digital literacy of older adults and associated factors is also necessary to foster the development of person-centered digital health care services and educational interventions for older adults in the digital society.

## Appendix

Multimedia Appendix 1 Interitem correlation matrix.

Multimedia Appendix 2 Scores for each item and the results of exploratory factor analysis.

Multimedia Appendix 3 Multiple indices for the measurement invariance of the Everyday Digital Literacy Questionnaire across the sex, age, and education groups.

Multimedia Appendix 4 The Korean version of the Everyday Digital Literacy Questionnaire, 22 items.

## Abbreviations

AVE: average variance extracted
CFA: confirmatory factor analysis
CFI: comparative fit index
CR: composite reliability
DigComp: Digital Competence
EDLQ: Everyday Digital Literacy Questionnaire
EFA: exploratory factor analysis
eHEALS: eHealth Literacy Scale
I-CVI: item-level content validity index
KMO: Kaiser-Meyer-Olkin
RMSEA: root-mean-square error of approximation
S-CVI: scale’s content validity index
SRMR: standardized root-mean-square residual

## Data Availability

The data sets generated and analyzed during this study are available from the corresponding author on reasonable request.
