# Supplementary material for: Everyday Digital Literacy Questionnaire for Older Adults: Instrument Development and Validation Study
Source: J Med Internet Res. 2023 Dec 14;25:e51616. doi: 10.2196/51616 (PMC10755654; doi:10.2196/51616)
Supplement: Multimedia Appendix 1 [file jmir_v25i1e51616_app1.pdf]

Interitem correlation matrix

	Q1	Q2	Q3	Q4	Q5	Q6	Q7	Q8	Q9	Q10	Q11	Q12	Q13	Q14	Q15	Q16	Q17	Q18	Q19	Q20	Q21	Q22	Q23	Q24	Q25	Q26	Q27	Q28	Q29	Q30
Q1	1																													
Q2	0.85	1																												
Q3	0.83	0.89	1																											
Q4	0.73	0.69	0.68	1																										
Q5	0.79	0.74	0.74	0.80	1																									
Q6	0.78	0.72	0.72	0.62	0.76	1																								
Q7	0.71	0.63	0.65	0.75	0.79	0.68	1																							
Q8	0.69	0.69	0.70	0.71	0.73	0.65	0.74	1																						
Q9	0.75	0.71	0.71	0.71	0.75	0.71	0.71	0.79	1																					
Q10	0.73	0.68	0.71	0.69	0.73	0.72	0.68	0.71	0.83	1																				
Q11	0.63	0.60	0.65	0.68	0.67	0.59	0.72	0.75	0.79	0.79	1																			
Q12	0.64	0.62	0.64	0.55	0.67	0.71	0.57	0.57	0.62	0.65	0.57	1																		
Q13	0.66	0.59	0.61	0.52	0.66	0.77	0.59	0.59	0.62	0.67	0.55	0.75	1																	
Q14	0.60	0.54	0.55	0.67	0.65	0.56	0.67	0.67	0.64	0.65	0.69	0.51	0.55	1																
Q15	0.70	0.68	0.67	0.69	0.68	0.67	0.65	0.69	0.73	0.79	0.74	0.63	0.66	0.73	1															
Q16	0.71	0.67	0.67	0.67	0.73	0.71	0.65	0.70	0.73	0.75	0.70	0.67	0.71	0.71	0.83	1														
Q17	0.58	0.53	0.55	0.71	0.65	0.49	0.63	0.64	0.63	0.63	0.70	0.48	0.45	0.77	0.70	0.66	1													
Q18	0.58	0.55	0.55	0.65	0.60	0.49	0.59	0.64	0.68	0.63	0.70	0.44	0.43	0.77	0.68	0.65	0.85	1												
Q19	0.64	0.61	0.59	0.58	0.66	0.65	0.59	0.63	0.66	0.66	0.59	0.61	0.63	0.57	0.69	0.72	0.55	0.59	1											
Q20	0.65	0.63	0.61	0.58	0.66	0.68	0.59	0.64	0.67	0.70	0.61	0.68	0.66	0.61	0.72	0.74	0.57	0.57	0.85	1										
Q21	0.71	0.66	0.68	0.71	0.73	0.66	0.71	0.71	0.78	0.76	0.74	0.62	0.62	0.69	0.78	0.76	0.70	0.71	0.73	0.76	1									
Q22	0.70	0.64	0.64	0.65	0.67	0.66	0.67	0.69	0.76	0.73	0.72	0.58	0.60	0.71	0.75	0.74	0.66	0.68	0.69	0.73	0.88	1								
Q23	0.72	0.68	0.70	0.60	0.72	0.73	0.62	0.68	0.73	0.73	0.62	0.70	0.73	0.56	0.72	0.76	0.56	0.55	0.76	0.80	0.79	0.77	1							
Q24	0.71	0.67	0.68	0.66	0.74	0.69	0.68	0.72	0.75	0.73	0.67	0.64	0.67	0.66	0.74	0.77	0.63	0.65	0.75	0.77	0.80	0.77	0.84	1						
Q25	0.70	0.67	0.66	0.66	0.74	0.70	0.69	0.71	0.76	0.76	0.69	0.66	0.69	0.63	0.75	0.76	0.62	0.63	0.76	0.79	0.80	0.79	0.84	0.86	1					
Q26	0.60	0.58	0.57	0.55	0.65	0.66	0.55	0.63	0.65	0.64	0.54	0.64	0.67	0.54	0.66	0.69	0.52	0.52	0.76	0.77	0.71	0.69	0.77	0.75	0.79	1				
Q27	0.60	0.56	0.56	0.54	0.62	0.65	0.56	0.62	0.62	0.63	0.56	0.63	0.68	0.53	0.68	0.68	0.53	0.52	0.75	0.76	0.70	0.67	0.78	0.74	0.78	0.89	1			
Q28	0.64	0.60	0.60	0.70	0.64	0.55	0.65	0.66	0.69	0.67	0.71	0.51	0.51	0.69	0.73	0.69	0.77	0.75	0.64	0.66	0.74	0.74	0.65	0.73	0.74	0.62	0.63	1		
Q29	0.62	0.58	0.59	0.67	0.62	0.53	0.63	0.67	0.69	0.65	0.69	0.49	0.49	0.69	0.72	0.69	0.76	0.76	0.61	0.62	0.74	0.75	0.65	0.74	0.72	0.61	0.60	0.91	1	
Q30	0.59	0.62	0.62	0.58	0.59	0.58	0.54	0.63	0.66	0.64	0.63	0.61	0.57	0.57	0.73	0.71	0.62	0.64	0.65	0.68	0.73	0.72	0.74	0.74	0.72	0.67	0.67	0.75	0.76	1
