# Supplementary material for: Everyday Digital Literacy Questionnaire for Older Adults: Instrument Development and Validation Study
Source: J Med Internet Res. 2023 Dec 14;25:e51616. doi: 10.2196/51616 (PMC10755654; doi:10.2196/51616)
Supplement: Multimedia Appendix 2 [file jmir_v25i1e51616_app2.pdf]

Scores for each item and the results of exploratory factor analysis

Items (abbreviated)	Mean (SD)	1st EFA			2nd EFA			3rd EFA			4th EFA			5th EFA			6th EFA		
		F1	F2	F3	F1	F2	F3	F1	F2	F3	F1	F2	F3	F1	F2	F3	F1	F2	F3
1. Find information I need on the Internet	3.15 (1.35)		0.89			0.90			0.91			0.93			0.93			0.93	
2. Judge whether the information from the Internet is reliable or not	2.95 (1.31)		0.80			0.80			0.83			0.84			0.84			0.84	
3. Discern whether the information found on the Internet is for commercial purpose [a]	2.88 (1.31)																		
4. Transfer documents, photos, or video files from one device to another	2.50 (1.31)		0.70	0.35		0.70	0.36		0.70	0.36		0.70	0.34		0.70	0.34		0.70	0.34
5. Save Internet documents, photos, or video files you find	2.80 (1.37)		0.83			0.83			0.83			0.84			0.83			0.83	
6. Exchange messages, photos, and video files through a social networking service	3.13 (1.38)		0.80			0.79			0.77			0.76			0.76			0.76	
7. Exchange documents, photos, or video files via an email	2.59 (1.42)		0.74			0.73			0.72			0.72			0.72			0.72	
8. Participate in video calls or conferences using digital devices	2.49 (1.38)		0.55			0.53			0.53			0.54			0.54			0.54	
9. Express my opinion of “like/dislike” on others’ posts	2.60 (1.40)		0.58			0.56			0.56			0.57			0.57			0.57	
10. Comment on others’ posts	2.64 (1.42)		0.56			0.53			0.52			0.52			0.53			0.53	

11. Participate in debates, opinion surveys, etc. using digital devices	2.26 (1.26)		0.45	0.45															
12. Respect each other in digital communication	3.09 (1.39)	0.43	0.50		0.44	0.49		0.45	0.46										
13. Take photos or videos using digital devices	3.09 (1.42)	0.48	0.51		0.49	0.50													
14. Create a document using digital devices	2.17 (1.27)			0.56			0.57			0.64			0.65			0.64			0.63
15. Upload Internet posts using digital devices	2.49 (1.36)	0.38		0.33	0.39		0.33	0.37		0.36	0.37		0.36						
16. Upload pictures or videos using digital devices	2.56 (1.40)	0.43	0.34		0.43	0.34		0.42	0.31		0.42	0.30		0.42	0.32				
17. Convert document formats using digital devices	1.95 (1.12)			0.75			0.76			0.83			0.85			0.85			0.84
18. Edit and post documents, photos, or videos created by someone else	1.95 (1.12)			0.77			0.78			0.84			0.85			0.85			0.85
19. Be aware of the behaviors that infringe copyright	2.60 (1.40)	0.76			0.75			0.76			0.76			0.76			0.76		
20. Protect copyright of the work from others	2.60 (1.39)	0.81			0.81			0.81			0.81			0.81			0.80		
21. Set device passwords for logging in/out	2.43 (1.36)	0.45		0.34	0.45		0.34	0.44		0.34	0.44		0.33	0.44		0.32	0.44		0.32
22. Change device passwords [a]	2.42 (1.35)																		
23. Delete files stored on the device	2.73 (1.45)	0.75			0.75			0.75			0.76			0.76			0.75		
24. Delete my history of Internet search	2.48 (1.38)	0.62			0.62			0.61			0.61			0.62			0.61		
25. Block spam or phishing attempts on the Internet	2.49 (1.41)	0.67			0.67			0.67			0.68			0.68			0.68		

26. Be aware of the physical side effects that can result from excessive device use	2.75 (1.41)	0.95			0.94			0.94			0.95			0.95			0.95		
27. Be aware of the mental side effects that can result from excessive device use	2.73 (1.39)	0.96			0.95			0.95			0.96			0.96			0.96		
28. Independently troubleshoot issues related to device/app installation [a]	2.09 (1.17)																		
29. Independently troubleshoot issues related to device/app operation	2.04 (1.14)	0.36		0.61	0.35		0.62	0.31		0.64	0.30		0.64	0.31		0.63	0.31		0.63
30. Know how to ask help when encountering issues during devices/app installation or operation	2.30 (1.29)	0.63		0.31	0.62		0.31	0.60		0.32	0.60		0.33	0.60		0.32	0.59		0.32
Percentage of variance explained		75.6%			75.7%			76.0%			76.7%			76.7%			77.0%		
Kaiser–Meyer–Olkin (KMO)		0.97			0.97			0.97			0.97			0.97			0.97		
Bartlett's Chi-square (df)		16892.2 (351)*			16158.8 (325)*			15494.9 (300)*			15011.5 (276)*			14198.0 (253)*			13504.4 (231)*		

Abbreviations: SD, standard deviation; EFA, exploratory factor analysis; F1, factor 1; F2, factor 2; F3, factor 3.

[a] Based on the inter-item correlation analysis, items 3, 22, and 28 were considered redundant and were deleted before EFA.

* *P*<.001
