# Supplementary material for: Everyday Digital Literacy Questionnaire for Older Adults: Instrument Development and Validation Study
Source: J Med Internet Res. 2023 Dec 14;25:e51616. doi: 10.2196/51616 (PMC10755654; doi:10.2196/51616)
Supplement: Multimedia Appendix 3 [file jmir_v25i1e51616_app3.pdf]

Multiple indices for the measurement invariance of the Everyday Digital Literacy Questionnaire across the sex, age, and education groups

Group	Invariance model	Chi-square (df)	CFI	RMSEA	SRMR	ΔCFI	ΔRMSEA	ΔSRMR
Sex								
	Configural	1939.403 (412)	0.873	0.121	0.066	-	-	-
	Metric	1945.753 (431)	0.874	0.118	0.067	0.001	-0.003	0.001
	Scalar	1945.753 (453)	0.876	0.114	0.067	0.002	-0.004	0.000
Age								
	Configural	1963.328 (412)	0.860	0.122	0.070	-	-	-
	Metric	1978.867 (431)	0.860	0.119	0.071	0.000	-0.003	0.001
	Scalar	1978.867 (453)	0.862	0.115	0.071	0.002	-0.004	0.000
Education								
	Configural	1910.061 (412)	0.850	0.120	0.075	-	-	-
	Metric	1923.784 (431)	0.851	0.117	0.077	0.001	-0.003	0.002
	Scalar	1923.784 (453)	0.853	0.113	0.077	0.002	-0.004	0.000

Abbreviations: CFI, comparative fit index; df, degrees of freedom; RMSEA, root mean square error of approximation; SRMR, standardized root mean square residual.
