# Supplementary material for: Everyday Digital Literacy Questionnaire for Older Adults: Instrument Development and Validation Study
Source: J Med Internet Res. 2023 Dec 14;25:e51616. doi: 10.2196/51616 (PMC10755654; doi:10.2196/51616)
Supplement: Multimedia Appendix 4 [file jmir_v25i1e51616_app4.pdf]

The Korean version of the Everyday Digital Literacy Questionnaire, 22 items

각 문항에서 현재 귀하의 경험과 의견을 가장 잘 반영한 것을 고르세요.

문항	전혀 그렇지 않다	별로 그렇지 않다	보통이다	대체로 그렇다	매우 그렇다
1. 나는 인터넷에서 필요한 정보를 찾을 수 있다.	①	②	③	④	⑤
2. 나는 인터넷에서 찾은 정보가 믿을만한 것인지 판단할 수 있다	①	②	③	④	⑤
3. 나는 디지털 기기 간에 문서, 사진 또는 동영상 등을 옮길 수 있다. (예: 스마트폰의 사진을 컴퓨터로 이동)	①	②	③	④	⑤
4. 나는 인터넷에서 찾은 문서, 사진 또는 동영상 등을 저장할 수 있다.	①	②	③	④	⑤
5. 나는 SNS를 이용하여 문자메시지, 사진 또는 동영상 등을 주고받을 수 있다. (SNS 예: 카카오톡, 페이스북 등)	①	②	③	④	⑤
6. 나는 이메일을 이용하여 문서, 사진 또는 동영상 등을 보내거나 받을 수 있다.	①	②	③	④	⑤
7. 나는 디지털 기기를 이용하여 영상통화 또는 화상회의에 참여할 수 있다.	①	②	③	④	⑤
8. 나는 타인의 글, 사진 또는 동영상에 "좋아요" 또는 "싫어요" 와 같은 의사표시를 할 수 있다. (예: 카카오톡, 유튜브, 온라인 카페 등)	①	②	③	④	⑤

9. 나는 타인의 글, 사진 또는 동영상에 댓글을 달 수 있다.<br>(예: 카카오톡, 유튜브, 온라인 카페 등)	①	②	③	④	⑤
10. 나는 디지털 기기를 이용하여 문서를 작성할 수 있다.	①	②	③	④	⑤
11. 나는 디지털 기기를 이용하여 문서 형식을 변환할 수 있다.<br>(예: 한글 문서를 PDF 문서로 변환)	①	②	③	④	⑤
12. 나는 타인이 만든 문서, 사진 또는 동영상을 편집하여 인터넷에 게시할 수 있다.	①	②	③	④	⑤
13. 나는 디지털 기기 또는 앱을 작동할 때 문제가 발생할 경우 스스로 해결할 수 있다.	①	②	③	④	⑤
14. 나는 타인이 만든 문서, 사진 또는 동영상을 인터넷에 허락받지 않고 올리는 일이 저작권을 침해하는 것임을 알고 있다.	①	②	③	④	⑤
15. 나는 새로운 문서, 사진 또는 동영상을 만들 때, 타인의 저작권을 보호한다.	①	②	③	④	⑤
16. 나는 디지털 기기에 비밀번호를 설정해서 로그인/로그아웃을 할 수 있다.	①	②	③	④	⑤
17. 나는 디지털 기기에 저장된 사진이나 동영상을 필요 시 삭제할 수 있다.	①	②	③	④	⑤
18. 나는 인터넷 검색 기록을 필요 시 삭제할 수 있다.	①	②	③	④	⑤
19. 나는 디지털 기기에서 스팸문자나 피싱문자와 같은 위험한 문자를 차단하는 방법을 알고 있다.	①	②	③	④	⑤

20. 나는 디지털 기기를 과도하게 사용할 경우 거북목, 손목 증후군과 같은 신체적 부작용이 발생할 수 있음을 알고 있다.	①	②	③	④	⑤
21. 나는 디지털 기기를 과도하게 사용할 경우 디지털 중독과 같은 정신적 부작용이 발생할 수 있음을 알고 있다.	①	②	③	④	⑤
22. 나는 디지털 기기 또는 앱을 설치하거나 작동할 때 발생한 문제를 스스로 해결할 수 없을 경우 어떻게 도움을 요청할지 알고 있다.	①	②	③	④	⑤
